# Supplementary material for: Soil organic carbon, physical fractions of the macro-organic matter, and soil stability relationship in lacustrine soils under banana crop
Source: PLoS One. 2021 Jul 9;16(7):e0254121. doi: 10.1371/journal.pone.0254121 (PMC8270212; doi:10.1371/journal.pone.0254121)

PLOS ONE

Soil organic carbon, physical fractions of the macro-organic matter, and soil stability relationship in lacustrine soils under banana crop

--Manuscript Draft--

**Manuscript Number:**	PONE-D-20-41023
**Article Type:**	Research Article
**Full Title:**	Soil organic carbon, physical fractions of the macro-organic matter, and soil stability relationship in lacustrine soils under banana crop
**Short Title:**	Physical fractions of the macro-organic matter in lacustrine soils
**Corresponding Author:**	Tatiana Rondon, M.S.<br>Corporacion Colombiana de Investigación Agropecuaria: AGROSAVIA<br>Rionegro, Antioquia COLOMBIA
**Keywords:**	aggregate size fraction; size-density fractionation; soil organic matter; water-stable aggregates
**Abstract:**	In order to evaluate the effect of the soil organic carbon (SOC) content and its distribution in the water-stable of soil aggregates (WAS), and the physical fractions of the macro-organic matter, a study was conducted in lacustrine soils under Banana (Musa AAA subgroup Cavendish) cv. 'Grand Nain' in Venezuela. Soil sampling was carried out in two batches differentiated by their production. A completely randomized design under a directed random sampling technique was carried out. In each condition, 12 composite samples were taken at depths 0-5 and 5-10 cm, respectively. WAS were separated into micro (<250 μm) and macro-aggregates (>250 μm). Also, physical fractionation by size-density of the macro-organic matter into light (LF), intermediate (IF), and heavy (HF) fraction using a silica gel solution, and SOC, were determined and correlated with banana yield and other agronomic traits. A major proportion of aggregates >250 μm were found in both condition and depths. Organic carbon within soil aggregates ranged between 29.7 and 35.3 g kg^{-1}. The HF was superior than IF and LF; however, its Carbon content was higher in the LF. The results allow inferring that the stability conferred to these soils is primarily associated with the presence of the snail, which shares the same size of the aggregates studied. High yields are associated with high carbon content in stable aggregates, as well as a higher content of carbon in the most labile fractions of macro-organic matter.
**Order of Authors:**	Tatiana Rondon, M.S.
	Rosa Mary Hernandez
	Manuel Guzman
**Additional Information:**	
**Question**	**Response**
**Financial Disclosure**<br><br>Enter a financial disclosure statement that describes the sources of funding for the work included in this submission. Review the submission guidelines for detailed requirements. View published research articles from *PLOS ONE* for specific examples.<br><br>This statement is required for submission and **will appear in the published article** if the submission is accepted. Please make sure it is accurate.	The author(s) received no specific funding for this work.

Powered by Editorial Manager® and ProduXion Manager® from Aries Systems Corporation

**Unfunded studies**<br>Enter: *The author(s) received no specific funding for this work.*<br><br>**Funded studies**<br>Enter a statement with the following details:<br>• Initials of the authors who received each award<br>• Grant numbers awarded to each author<br>• The full name of each funder<br>• URL of each funder website<br>• Did the sponsors or funders play any role in the study design, data collection and analysis, decision to publish, or preparation of the manuscript?<br>• **NO** - Include this sentence at the end of your statement: *The funders had no role in study design, data collection and analysis, decision to publish, or preparation of the manuscript.*<br>• **YES** - Specify the role(s) played.<br><br>* typeset	
**Competing Interests**<br><br>Use the instructions below to enter a competing interest statement for this submission. On behalf of all authors, disclose any competing interests that could be perceived to bias this work—acknowledging all financial support and any other relevant financial or non-financial competing interests.<br><br>This statement **will appear in the published article** if the submission is accepted. Please make sure it is accurate. View published research articles from *PLOS ONE* for specific examples.	The authors have declared that no competing interests exist.

**NO authors have competing interests**<br><br>Enter: *The authors have declared that no competing interests exist.*<br><br>**Authors with competing interests**<br><br>Enter competing interest details beginning with this statement:<br><br>*I have read the journal's policy and the authors of this manuscript have the following competing interests: [insert competing interests here]*<br><br>* typeset	
**Ethics Statement**<br><br>Enter an ethics statement for this submission. This statement is required if the study involved:<br><br>• Human participants<br>• Human specimens or tissue<br>• Vertebrate animals or cephalopods<br>• Vertebrate embryos or tissues<br>• Field research<br><br>Write "N/A" if the submission does not require an ethics statement.<br><br>General guidance is provided below. Consult the submission guidelines for detailed instructions. **Make sure that all information entered here is included in the Methods section of the manuscript.**	N/A

**Format for specific study types**<br><br>**Human Subject Research (involving human participants and/or tissue)**<br>• Give the name of the institutional review board or ethics committee that approved the study<br>• Include the approval number and/or a statement indicating approval of this research<br>• Indicate the form of consent obtained (written/oral) or the reason that consent was not obtained (e.g. the data were analyzed anonymously)<br><br>**Animal Research (involving vertebrate animals, embryos or tissues)**<br>• Provide the name of the Institutional Animal Care and Use Committee (IACUC) or other relevant ethics board that reviewed the study protocol, and indicate whether they approved this research or granted a formal waiver of ethical approval<br>• Include an approval number if one was obtained<br>• If the study involved *non-human primates*, add *additional details* about animal welfare and steps taken to ameliorate suffering<br>• If anesthesia, euthanasia, or any kind of animal sacrifice is part of the study, include briefly which substances and/or methods were applied<br><br>**Field Research**<br><br>Include the following details if this study involves the collection of plant, animal, or other materials from a natural setting:<br>• Field permit number<br>• Name of the institution or relevant body that granted permission	
**Data Availability**<br><br>Authors are required to make all data underlying the findings described fully available, without restriction, and from the time of publication. PLOS allows rare exceptions to address legal and ethical concerns. See the PLOS Data Policy and FAQ for detailed information.	Yes - all data are fully available without restriction

A Data Availability Statement describing where the data can be found is required at submission. Your answers to this question constitute the Data Availability Statement and **will be published in the article**, if accepted.<br><br>**Important:** Stating 'data available on request from the author' is not sufficient. If your data are only available upon request, select 'No' for the first question and explain your exceptional situation in the text box.<br><br>Do the authors confirm that all data underlying the findings described in their manuscript are fully available without restriction?	
**Describe where the data may be found in full sentences. If you are copying our sample text, replace any instances of XXX with the appropriate details.**<br><br>• If the data are **held or will be held in a public repository**, include URLs, accession numbers or DOIs. If this information will only be available after acceptance, indicate this by ticking the box below. For example: *All XXX files are available from the XXX database (accession number(s) XXX, XXX.)*.<br>• If the data are all contained **within the manuscript and/or Supporting Information files**, enter the following: *All relevant data are within the manuscript and its Supporting Information files.*<br>• If neither of these applies but you are able to provide **details of access elsewhere**, with or without limitations, please do so. For example:<br><br>*Data cannot be shared publicly because of [XXX]. Data are available from the XXX Institutional Data Access / Ethics Committee (contact via XXX) for researchers who meet the criteria for access to confidential data.*<br><br>*The data underlying the results presented in the study are available from (include the name of the third party*	All relevant data are within the paper and on Figshare via a http://dx.doi.org/10.6084/m9.figshare.13507050

and contact information or URL).<br>• This text is appropriate if the data are owned by a third party and authors do not have permission to share the data.<br><br>* typeset	
Additional data availability information:	Tick here if the URLs/accession numbers/DOIs will be available only after acceptance of the manuscript for publication so that we can ensure their inclusion before publication.

Manuscript

Click here to access/download;Manuscript;paperOMF.v1.PLOSONE.docx

Soil organic carbon, physical fractions of the macro-organic matter, and soil stability relationship in lacustrine soils under banana crop

Tatiana Rondon[1,2*], Rosa Mary Hernández[3], Manuel Guzman[2]

[1] Facultad de Agronomía, Universidad Central de Venezuela (UCV), Maracay, Aragua, Venezuela

[2] Centro de Investigación La Selva, Corporación Colombiana de Investigación Agropecuaria (AGROSAVIA), Rionegro, Antioquia, Colombia

[3] Instituto de Estudios Científicos y Tecnológicos (IDECYT), Universidad Nacional Experimental Simón Rodríguez (UNESR), Altos de El Cují, Miranda, Venezuela

* Corresponding author

Email: trondon@agrosavia.co (TR)

Abstract

In order to evaluate the effect of the soil organic carbon (SOC) content and its distribution in the water-stable of soil aggregates (WAS), and the physical fractions of the macro-organic matter, a study was conducted in lacustrine soils under Banana (*Musa* AAA subgroup Cavendish) cv. 'Grand Nain' in Venezuela. Soil sampling was carried out in two batches differentiated by their production. A completely randomized design under a directed random sampling technique was carried out. In each condition, 12 composite samples were taken at depths 0-5 and 5-10 cm, respectively. WAS were separated into micro (<250 µm) and macro-aggregates (>250 µm). Also, physical fractionation by size-density of the macro-organic matter into light (LF), intermediate (IF), and heavy (HF) fraction using a silica gel solution, and SOC, were determined and correlated with banana yield and other agronomic traits. A major proportion of aggregates >250 µm were found in both condition and depths. Organic carbon within soil aggregates ranged between 29.7 and 35.3 g kg^{-1}. The HF was superior than IF and LF; however, its Carbon content was higher in the LF. The results allow inferring that the stability conferred to these soils is primarily associated with the presence of the snail, which shares the same size of the aggregates studied. High yields are associated with high carbon content in stable aggregates, as well as a higher content of carbon in the most labile fractions of macro-organic matter.

Introduction

Agricultural land use and cropping systems have promoted an accelerated degradation of soils with important changes in their chemical, physical, and biological dynamics. In this scenario, continuous soil uses without proper agricultural practices affect

the sustainability of cropping systems and will be at risk at mid-term [1-3]. Once the soil environment has been interfered, the availability of nutrients such as nitrogen and phosphorus decreases, as well as the soil organic carbon (SOC) contents [4-5], even when the soil management carried out does not involve changes in the vegetation cover [6].

A stable soil structure is the most desirable soil characteristic for sustaining agricultural productivity and for preserving environmental quality [7]. Several soil properties are dependent on the dynamics of soil structure, which is usually defined such as the aggregates' ability to resist the separation and dispersion under soil moisture, anthropic action or other external factors. The aggregates are primarily formed by physical processes. Meanwhile, biological and chemical processes are mainly responsible for their stabilization [8,9]. Primary soil particles (sand, silt, and clay) are held together firmly, so they not disperse in water by cementing agents such as soil organic matter (SOM) contents, quantity and types of clay particles, iron and aluminum hydroxides and others [10,11]. However, clay flocculation is a pre-requisite for soil aggregation [12]. Soil aggregates are an important component of soil structure due to their importance in the protection of soil loss by erosion [13] and degradation [7]. In addition, aggregate formation in soils is an important process in SOC stabilization [14,15] isolating it from decomposers [16]. Arshad and Coen [17] proposed aggregate stability as one of the soil physical properties that can serve as an indicator of soil quality. According to Tisdall and Oades [18], aggregates are classified as micro-aggregates (<250 µm) and macro-aggregates (>250 µm). The stability of soil macro- and micro-aggregates are used to estimated soil erosion, soil erodibility and surface runoff [19-21].

Ma et al. [22] indicated that is necessary to understand the relationship between water-stable aggregates and soil fertility properties since macro-aggregates are destroyed easily with cultivation but the organic matter within is decomposed partially. Biological soil properties are an important fraction of soil quality, and are maintained or improved through conservation agriculture farming practices causing positive changes in SOC accumulation and microbial activity [23]. The SOM has an important role as source and sink because its interaction with several soil variables such as soil aeration, moisture retention, nitrogen fixation and aggregate formation [24-26]. Chien et al. [27] indicated the influence of SOM over different soil processes and the importance to study the quantity and type of SOM.

In addition, the study of carbon pools in the soil aggregates allows knowing the dynamics of soil carbon sequestration and mineralization in the aggregates [28], as well as it influences on crop development and yields, eventually [29]. Several studies suggest a fractionation of SOM as an effective procedure to obtain more information on SOM types and its distribution in the soil matrix [30-32]. Several techniques to measure the size and turnover of SOM pools have been developed and used to separate SOM into labile and recalcitrant pools. This classification is based on the relative susceptibility to biological decomposition [31,33]. These methods rely on chemical, physical, or biological separation [34]. However, physical fractionation is the most used by the scientific community for being considered less destructive than chemical methods, and since it is relates better to the structure and function of SOM *in situ* [35,36]. Physical fractionation has been applied to determine the association of SOM with primary particles and to quantify the amount of particulate organic matter between and within soil aggregates [37,38]. Fractionation by density is based on submersion of soil samples into inorganic salt solutions with a specific

density between 1.6 to 2.2 g cm^{-3} and limited sample dispersion [39]. This process separates the light and heavy fractions, where the light is considered more labile and with a density lower than the soil minerals. Meanwhile, the heavy fraction is assumed to be enriched in decomposition products stabilized onto the surface of clay or silt particles [31] or within soil micro-aggregates, making it more resistant to microbial degradation and with a higher specific density due to its intimate association with soil minerals.

In this research, our objective was to study the influence of soil organic matter, with special emphasis on the role played by the SOM fractions on banana yield and its components. To do this, we i) classified water-stables of soil aggregates, ii) fractioned SOM using a size-density method, iii) determined the COM contents in each fraction, and iv) analyzed the relationship among SOM, stability, and banana yield.

Materials and methods

Site description

Soil samples were taken in January 2009 in a commercial banana plantation with cv. 'Grand Nain' (*Musa* AAA, subgroup Cavendish) located at Lake Valencia basin, Venezuela (10°8′33″ N, 67°35′3″ W), chosen for this study due to its high productivity standards focused in exportation markets. Lake Valencia basin accounts more than 17,000 hectares under agriculture, where banana, sugarcane, and short-cycle vegetable are the major crops grown in the area. The sampling site was in lacustrine soils characterized by a high-water table and the presence of a carbonitic soil layer with abundant snail shells and more than 40% of $CaCO_3$. The soil was classified as Entisol, Mollic Ustifluvents, sandy loam, mixed,

calcareous, isohyperthermic according to USDA [40] keys of soil taxonomy, and was placed in a Quaternary terrace. Soils were moderate well-drained to well-drained. An initial chemical and physical profile of soils studied were reported by Sapucky [41] and Cruz [42] and are listed in Table 1. Altitude fluctuated between 410 and 440 m a.s.l. in the sampling area with a soil slope of ≤ 3%. The climatic classification according to Köppen was Tropical Dry (Aw). Long-term climate data indicated annual average precipitation of 1028 mm, with a unimodal pattern of six months of rainfalls distributed between mid-May and October. Also, the location had average evaporation of 1500 mm, a relative humidity of 67%, and temperature fluctuation between 17.5 and 28.0 °C.

Table 1. Soil properties of sampling sites studied

	**Condition 1**		**Condition 2**	
**Property**	**0-5 cm**	**5-10 cm**	**0-5 cm**	**5-10 cm**
Textural class	Silt loam	Silt loam	Loam	Loam
Sand (%)	8.80	9.10	7.20	7.90
Silt (%)	73.3	75.2	69.0	68.3
Clay (%)	18.8	15.7	23.1	24.5
Bulk density (g cm^{-3})	1.15	1.10	1.00	1.00
Soil pH (1:1)	7.73	7.77	7.84	7.78
Soil electrical conductivity (dS m^{-1})	0.87	0.78	1.62	2.10
Total soil porosity (%)	57.7	58.0	60.1	59.0
Soil Organic Matter (%)	4.42	4.92	5.91	4.89
Saturated Hydraulic Conductivity (Ks) (cm h^{-1})	1.69	3.89	6.56	6.55

Penetration resistance (kPa cm^{-1})	0.41	0.35	0.38	0.32

Note: adapted from Sapucky (2007) and Cruz (2011)

Soil sampling procedure and experimental design

The banana plantation studied presents marked differences in fruit yield among its plots and were divided according to an arbitrary, yet parsimonious, principle: (*i*) yield ≤28 t ha^{-1} (Condition 1), and (*ii*) yield ≥28 t ha^{-1} (Condition 2). This principle is based on the average banana fruit yield in Venezuela (~28 t ha^{-1}). Representative plots of both conditions were selected for this study, following homogeneous selection criteria as cultivar, a second cycle of plantation, agricultural practices, and physical and chemical soil conditions. A completely randomized design was used in combination with a simple random sampling technique with 6 replications, targeted in plants at the flowering stage. Each experimental unit or plot size was of 400 m^2. At ‘Condition 1’ and ‘Condition 2’, any litter layer was removed before soil sampling, and sampling was done in the base of the parent plant and close to the following sucker. All plants selected for soil sampling were at the starting flowering stage. The choice of sampling depth of 10 cm was based on the fact that this soil depth exhibits higher biological activity and the labile fraction of SOM.

Soils cores were taken with a shovel from the upper 10 cm and were divided into 2 depth increments: 0-5 cm and 5-10 cm. In both conditions, 3 kg of composite soil samples were collected from 0-5 and 5-10 cm depths. Each composite sample derived from 12 sub-samples collected in different points on the plot; all were well mixed in a bucket. From the soil extracted with the shovel, only the central part was recovered to avid collecting

aggregates disturbed by sampling. A hand-held GPS device with 0.5 m precision was used to georeferenced the location of sampling points.

Soil and crop variables evaluated

Composite soil samples were air-dried, visible plant roots removed and grounded to pass through a 2.0-mm sieve. Soil samples were stored in hermetic plastic bags until analyses. All laboratory analyses were replicated three times for each sample. The water-stables of soil aggregates (WSA) were determined using an Eijkelkamp wet sieving apparatus (Eijkelkamp Agrisearch Equipment, Glesbeek, The Netherlands) following the description developed by Florentino [43] with modifications. Sub-samples of 20 g of 1-2 mm air-dried aggregates were pre-wetted by capillary action using a paper napkin with distilled water, by 5 minutes. Later, pre-wetted sub-samples were put into the Eijkelkamp apparatus and wet sieving by 3 minutes at a constant speed. The amount of WSA was determined based on soil material kept on the 250 µm mesh, classified as soil macroaggregates (>250 µm) and microaggregates (<250 µm), respectively. The macro and microaggregates were dry in an oven at 105 °C by 24 h and weighted.

Size-density fractionation of SOM was conducted using a silica suspension (LUDOX) adjusted to 1.37 g cm^{-3} as described by Meijboom et al. [44] with modifications (Fig 1). In short, 500 g of air-dried 2 mm sieved soil was weighed into 3 L beaker, 2 L of deionized water was added and vigorously shaken, with the aim to separate particulate organic matter from soil mineral fraction. The suspension was filtered through superimposed sieves of 250 and 150 µm, respectively located at top and bottom, and washed carefully to separate by size. A jet of water was applied into soil material retained on the sieves, washed,

and swirled to separate macro-organic matter from mineral fraction by decanting. Swirling and decanting were repeated several times until no floating materials remained using the 150 µm sieve. The organic material collected in the 150 µm sieve was submerged in the LUDOX suspension adjusted to 1.13 g cm^{-3} by ten minutes. The floating fraction was collected and correspond to the light fraction (LF). The remaining fraction in the sieve was placed in LUDOX suspension adjusted to 1.37 g cm^{-3} by ten minutes. The new floating fraction was an intermediate fraction (IF) and the non-floating fraction corresponded to the heavy fraction (HF). All three fractions collected were washed with deionized water and dried in an oven at 50 °C until to constant weight.

Fig 1. Schematic representation of size-density fractionation of SOM

Soil organic carbon (SOC) by each WSA (macro and microaggregate), and SOM fractions (LLF, LIF and LHF) were determined using wet digestion method according to Walkley-Black [45] and modified by Heanes [46], using potassium dichromate ($K_2Cr_2O_7$) and concentrated sulfuric acid (H_2SO_4).

At harvesting, in the same plots where soil samples were collected, banana crop traits were recorded such as yield (Y, t ha^{-1} $year^{-1}$), pseudostem diameter (PD), determined by the circumference at 100 cm from the ground; plant height (PH), from the base of pseudostem to the point of bunch emergence expressed in cm; number of hands per bunch (HB), and bunch weight (BW) expressed in kg.

Data analyses

The normality of data was assessed using Shapiro-Wilk test ($p \leq 0.05$). A one-way analysis of variance (ANOVA) was used to analyze the data. Means were separated using Tukey test at $p \leq 0.05$ if there was a significant site effect. Correlation analysis by Pearson was used to determine the relationship between each pair of traits evaluated, using a statistical significance at the $\alpha < 0.05$ level.

Results

Water-stables of soil aggregates (WSA)

Significant differences in WSAs were observed between the conditions studied (Fig 2). In both examined depths, the proportion of >250 µm aggregate fraction was higher in condition 2, indicating that these soils were composed mainly of macro-aggregates. Water stable >250 µm aggregates were 77.41% and 89.25% for condition 1 and 2 at 0-5 cm, respectively; meanwhile, 75.27 and 84.23% was found at 5-10 cm. In contrast, a higher proportion of <250 µm aggregate was found in condition 1, with more than 20 %. The < 250 µm aggregate size range was low in both depths, with values between 7.98 (condition 2, depth 0-5 cm) and 23.29 % (condition 1, depth 5-10 cm). Both conditions were stable in structural terms; however, the condition 1 showed a higher percent of aggregates associated with the micro-aggregates (<250 µm) than condition 2.

Fig 2. Distribution by the size of water-stables aggregates at two soil depths in lacustrine soil studied. Barplot (a) and (b) represent >250 µm aggregates at 0-5 and 5-10 cm, respectively. Correspondingly, barplot (c) and (d) represent <250 µm aggregates at 0-5 and

5-10 cm, respectively. Different letters indicate statistically significant differences at the 0.05 level by Tukey's post-hoc test.

Organic carbon contents within soil aggregates

OC contents within soil aggregates showed highly significant differences ($p \leq 0.01$) among conditions studied at depth and size-aggregates level (Fig 3). In soil aggregates superior to 250 µm, the OC was superior in the condition 1 to both soil depths, with values of 32.7 and 29.7 g kg^{-1}, to 0-5 and 5-10 cm, respectively. On the other hand, in aggregates <250 µm the OC content was superior in the condition 2 with values of 35.3 and 32.3 g kg^{-1}, to 0-5 and 5-10 cm, respectively. In both conditions, a decrease was observed in the OC content at deeper soil layers.

Fig 3. Content of organic carbon associated with soil macro (>250 µm) and micro-aggregated (<250 µm) at two soil depths in lacustrine soil studied. Barplot (a) and (b) represent >250 µm aggregates at 0-5 and 5-10 cm, respectively. Correspondingly, barplot (c) and (d) represent <250 µm aggregates at 0-5 and 5-10 cm, respectively. Different letters indicate statistically significant differences at the 0.05 level by Tukey's post-hoc test.

Size-density fractionation of macro-organic matter (MOM)

Physical fractionation of LF, IF, and HF is showed in Table 2. Highly significant differences ($p \leq 0.01$) for all fractions in both soil depths were observed. 'Condition 2' showed higher values than 'Condition 1' for all fractions at 0-5 and 5-10 cm. Across soil depths and conditions, values ranged between 0.06-0.49, 0.14-1.50, and 0.45-2.93 g kg^{-1}, to LF, IF and

HF, respectively. In general, HF was superior to the other fractions, showing a fraction decreased in the order HF > IF > LF, for the soils studied.

Table 2. Distribution (g kg^{-1}) of macro-organic fractions studied

**Soil depth (cm)**		**LF (g kg^{-1})**	**IF (g kg^{-1})**	**HF (g kg^{-1})**
0-5	Condition 1	0.15±0.05b	0.52±0.25b	1.27±0.38b
	Condition 2	0.44±0.15a	1.50±0.40a	2.93±0.28a
5-10	Condition 1	0.06±0.03b	0.14±0.05b	0.45±0.10b
	Condition 2	0.49±0.17a	0.79±0.22a	1.98±0.25a

Different letters indicate statistically significant differences at the 0.01 level by Tukey's post-hoc test

Soil organic carbon (SOC) contents and its distribution in SOM fractions

Significant differences in the content of C in the different SOM fractions were found between conditions in both depths (Fig 4). The content of C was generally lower at 5-10 depth, except in the light fraction at 0-5 cm. In both depths, the C content decreased across the fractions in the order LF > IM > HF. At 0-5 cm depth, mean values of 21.7, 18.2 and 10.9 g C kg^{-1} soil were found to LF, IM, and HR, respectively, across conditions; meanwhile, at 5-10 cm depth values of Carbon (g kg^{-1} soil) were 26.7 (LF), 14.1 (IF) and 8.1 (HF). Only in

the LF, an increasing trend in C content was observed at deeper soil depths. IF and HF showed a decrease in C content for both conditions.

Fig 4. Organic carbon (g kg^{-1} soil) associated with the different macro-organic matter fractions. Light, intermediate, and heavy fraction at (a) 0-5 and (b) 5-10 cm, respectively. Different letters indicate statistically significant differences at the 0.05 level by Tukey's post-hoc test.

Banana yield and agronomic variable evaluated

Yield and other agronomic variables showed a significant difference at 0.05 level among conditions studied (Table 3), where all evaluated variables were superior in condition 2. Yield average was 67.6 t ha^{-1} year^{-1} across the condition studied, where the 'Condition 2' had a higher yield than 'Condition 1', with 74.61 and 60.48 t ha^{-1} year^{-1}, respectively, representing an increase in yield of 23.4%. Traits such as plant height, pseudostem diameter, hands per bunch and bunch weight are used as an indicator of vigor in a banana plantation. Plant height was 44 cm superior in condition 2 than condition 1. Pseudostem diameter presented an average of 82.9 cm. The number of hands per bunch showed a difference of 1.85 between the conditions studied; meanwhile, the bunch weight ranged between 33.61 and 36.59 kg among conditions.

Table 3. Yield and agronomic variables evaluated in this study

	**Y (t ha^{-1} year^{-1})**	**PH (cm)**	**PD (cm)**	**HB**	**BW (kg)**

Condition 1	60.48 b	282 b	75.3 b	9.5 b	33.61 b
Condition 2	74.61 a	326 a	90.6 a	11.4 a	36.59 a

Y: yield, PH: plant height, PD: pseudostem diameter, HB: hands per bunch, BW: bunch weight. Different letters indicate statistically significant differences at the 0.05 level by Tukey's post-hoc test

Relationship among soil aggregates, size-fraction, and content of organic carbon, and agronomic traits

To identify the relationship between pairs of the variables studied, a Pearson correlation analysis was performed (Fig 5). The content of Carbon in the macro-aggregates (CMA) was highly and negatively correlated with the CIF and CHF, with values of $r = -0.69$ and $r = -0.74$, respectively. However, to both variables, a positive correlation was found in the content of C in the soil micro-aggregates (CMI). The Carbon content in the different fractions of SOM: light (CLF), intermediate (CIF), and heavy (CHF) were evaluated. A high relationship between the different fractions of the SOM and the WSA was found. A high positive correlation ($p \leq 0.01$) was found among yield and PH, OC, HB, BW, PD, CLF, and CMA; meanwhile, a negative correlation was found among CIF, CHF, and CMI. PH also had a significant correlation with all variables studied, positive in all cases except for CIF, CHF, and CMI, indicating that these variables did not a positive contribution on yield. Significantly positive values ($p \leq 0.05$) were found between OC and HB, and among PD, BW, CLF and PH. Vigor traits such as PH, HB, PD, BW, and Y were correlated positively. In addition, a

positive correlation was found between the CMA and all variables associated with the vigor of the plantation.

Fig 5. Heatmap of correlation coefficient among soil aggregates, size-fraction and content of organic carbon, and agronomic traits. Pearson correlation is showed between variables described in this study. Yield (Y), pseudostem diameter (PD), plant height (PH), number of hands per bunch (HB), bunch weight (BW), carbon content at light fraction (CLF), carbon content at intermediate fraction (CIF), carbon content at heavy fraction (CHF), carbon content at macroaggregates (CMA), carbon content at microaggregates (CMI).

Discussion

In the current study, the aggregate distribution shows that more than 70 % of the soil was composed of macro-aggregates (>250 µm) in both conditions. Our results further reveal that condition 2 has a superior proportion of aggregates >250 µm at depths of 0-5 and 5-10 cm. In the soil, the influence of the aggregate size over other factors such as water retention, and water and air movement are crucial. This allows establishing relations among pore distribution, erosion, water movement, gaseous exchange, and crop productivity [47,48]. In the initial survey (Table 1), the total porosity range between 57.7 and 60.1 %, being higher in the condition 2, as well as found in the soil macro-aggregates distribution (Fig 2). This suggests a better aggregate stability of the soil. When the proportion of large to small aggregates increases, soil quality generally increases; however, this is highly dependent of SOM and biological activity in the soil.

The storage of SOC at soil aggregates differs from the behavior of WSA proportion. At condition 2, higher Carbon contents were found in soil micro-aggregates (<250 µm). In addition, a better structural stability and a SOC stable or physically protected to the action of external agents such as microorganisms, raindrop impact or anthropogenic action. The results suggest that the high content of SOM (5.91 %) in this condition play an important role as a cementing agent in the WSA and SOC. This matches the observations made by Tisdall and Oades [18] and Cabria et al. [49]. The soil aggregate stability may be under the influence of cementing or binding agents, which may be inorganic, organo-mineral association or organic, as is suggested by Tisdall and Oades [18]. High contents of SOC were found associated with recalcitrant or stable forms such as soil aggregates size lower than 250 µm, indicating a high activity of soil microorganisms, which are highly efficient in the decomposition of SOM. Our results further signify a high relation between SOC in aggregates with yield and vigor traits such as plant height and pseudostem diameter (Fig 5). In contrast, condition 1 shows lower SOC content, which may be a reason for lower structural stability. Also, this condition presents low values of OC associated with less recalcitrant form such as aggregate size greater than 250 µm, as a consequence of the deficient activity of soil microorganisms. Jastrow and Miller [50] suggest that the stability of micro-aggregates is influenced by the forces with which the particulate fraction of OM is able to suck clays, and inorganic and organic compounds, microbial residues, and organic colloids.

The MOM fractionation by size and density suggest that MOM tends to be heavy due to a rapid transformation of organic residues (inputs) into humified forms. In both conditions, the distribution of macro-organic fraction follow the order HF > IF > LF, at the soil depths studied. Condition 2 shows a faster and more efficient decomposition of residues by the

microorganisms. This result agrees with the report by Christensen [39], Zagal et al. [51] and Caballero [52], who indicate that the LF does not exceed 4 g kg^{-1} in cultivated soils. Different studies also specify that the LF is sensitive to soil management practices [30,53,54]. This occurs because the LF is regarded as highly labile, composed mainly by organic matter that still does not form complexes with minerals of the soil [55]. In this sense, Caballero [52], using LUDOX as fraction separator, discover that the proportion of each fraction, is mainly due to the land-use and OC dynamics, in special at soils under crops by the microbial necromass contributions and the level of recalcitrant compounds.

The C content decreased across the MOM fractions in the order LF > IM > HF, but was associated mainly with the LF and IF, to both conditions and soil depths studied. These results suggest that the OC with more content in the MOM is unstable and easy to decompose. Six et al. [56] suggest a relation between the loss of organic C, N and structural stability with an increase of soil cultivation. A decreased of macro-aggregates with high C content and an increase of micro-aggregates depleted in C is considered as one of the most important reasons for the loss of SOM [56-58]. The content of C in all fractions was correlated with the examined crop traits. However, high contents of C in the recalcitrant fractions of SOM such as HF, showed a detrimental in the vigor of the studied banana plantation (Fig 5).

The complexity of MOM fractions varies according to its size; thus, smaller fractions are related to a higher rate of recalcitrance, complexity or resilience in the soil [59]. In general, banana plantations in Venezuela use as organic matter inputs from any tissues or leaf blades of the same crop, and organic compost as a complement. These inputs are labile and are quickly decomposed by the microorganisms, leading to more stable forms of MOM such as the IF and HF. Balesdent et al. [60] indicated the importance of the fractions of SOM,

given its role on the C dynamics on cultivated soils, including fractions called 'inert' or humus, even organic residues at the same level of importance. With this, a comprehensive approach is created including SOM quality and the transformation of organic fractions that involves both the micro-environment and the substrate where the interactions occur. A balance of the fractions of SOM is important because it works as an indicator of 'organic composition', where physical, chemical and soil management cause modifications of SOM [61].

The soils studied are lacustrine, with a high content of snail shells in its structure. This characteristic was present in the micro and macro-aggregates of the soil, as well in the physical fractions of SOM. Thus, the soil variables studied could be overestimated by the snail shell presence. This consideration is based on the presumption that the snail shell is part of the soil matrix. It is not considered as a soil aggregate even if it behaves similarly, regarding its conceding structure and porous space; but is neither considered as MOM. However, the analyses of the variables studied are based on weight and density, so its presence is necessarily considered.

Conclusions

The distribution of aggregates and its stability were different between the soil conditions studied. The stability of the soil aggregates was higher under the 'Condition 2', due to a major proportion of soil macro-aggregates (>250 µm) at both studied depths. Also, this condition showed a high content of Carbon associated in aggregates < 250 µm, contributing to an increase of the OC stability, and improving the soil-plant system. The high content of Carbon at macro-aggregates showed a positive effect on banana yield and other

agronomic variables evaluated. The physical fractions of SOM revealed a decrease in its proportions in the order HF > IF > LF. However, the dynamic of Carbon was inverted in the order CLF > CIF > CHF, facilitating the presence of Carbon into the labile fraction of MOM. The results indicate that the management of organic fertilization in these soils must be carried out with organic sources that promote high Carbon available levels in the labile soil fractions. Future research should focus in integrating approaches that combine chemical parameters in the soil and banana plants to evaluate this combination in the light of the soil organic matter.

Acknowledgments

The authors thanks to 'Finca Agropecuaria Punta Larga' and the technical personnel of Laboratory of Soils of Universidad Central de Venezuela and Universidad Simón Rodríguez.

References

1. Burbano-Orjuela H. El suelo y su relación con los servicios ecosistémicos y la seguridad alimentaria. Rev Cienc Agr. 2016; 32: 117-124. http://dx.doi.org/10.22267/rcia.163302.58
2. Kalu S, Koirala M, Khadka U, Anup R. Soil quality assessment for different land use in the panchase area of western Nepal. International Journal of Environmental Protection. 2016; 5: 38–43. http://doi.org/10.5963/IJEP0501006
3. Paz-Kagan T, Shachak M, Zaady E, Karnieli A. Evaluation of ecosystem responses to land-use change using soil quality and primary productivity in a semi-arid area, Israel. Agr Ecosyst Environ. 2014; 193: 9-24. http://doi.org/10.1016/j.agee.2014.04.024

4. Fernández P, Acevedo D, Villanueva A, Uribe G. Estado de los elementos químicos esenciales en suelos de los sistemas natural, agroforestal y de monocultivo. Revista Mexicana de Ciencias Forestales. 2016; 7: 65-77
5. Wang W, Wang C, Sardans J, Min Q, Zeng C, Tong C, et al. Agricultural land use decouples soil nutrient cycles in a subtropical riparian wetland in China. Catena. 2015; 133: 171-178. https://doi.org/10.1016/j.catena.2015.05.003
6. Smith P, House J, Bustamante M, Sobocká J, Harper R, Pan G, et al. Global change pressures on soils from land use and management. Glob Change Biol. 2016; 22: 1008-1028. https://doi.org/10.1111/gcb.13068
7. Amézketa E. Soil aggregate stability: a review. J Sustain Agric. 1999; 14: 83–151. https://doi.org/10.1300/J064v14n02_08
8. Allison FE. Soil aggregation-some facts and fallacies as seen by a microbiologist. Soil Sci. 1968; 106: 136–143
9. Lynch JM, Bragg E. Microorganisms and soil aggregate stability. In: Stewart BA, editor. Advances in Soil Science, vol 2. Springer; 1985.pp. 133–171. https://doi.org/10.1007/978-1-4612-5088-3_3
10. Fernández L. Aplicación del índice de estabilidad estructural de Pieri (1995) a suelos montañosos de Venezuela. Terra. 2014; 30: 143–153
11. Pulido M, Gabriels D, Cornelis W, Lobo D. Comparing aggregate stability tests for soil physical quality indicators. Land Degrad Dev. 2013; 26: 843–852. https://doi.org/10.1002/ldr.2225
12. Dexter AR. Advances in characterization of soil structure. Soil Tillage Res. 1998; 11: 199-238. https://doi.org/10.1016/0167-1987(88)90002-5

13. Moreno-de las Heras M. Development of soil physical structure and biological functionality in mining spoils affected by soil erosion in a Mediterranean continental environment. Geoderma. 2009; 149: 249–256. https://doi.org/10.1016/j.geoderma.2008.12.003

14. Alvaro-Fuentes J, Cantero-Martinez C, Lopez MV, Paustian K, Denef K, Stewart CE, et al. Soil aggregation and soil organic carbon stabilization: effects of management in semiarid Mediterranean agroecosystems. Soil Sci Soc Am J. 2009; 73: 1519–1529. https://doi.org/10.2136/sssaj2008.0333

15. Lützow Mv, Kögel-Knabner I, Ekschmitt K, Matzner E, Guggenberger G, Marschner B, et al. Stabilization of organic matter in temperate soils: mechanisms and their relevance under different soil conditions - a review. Eur J Soil Sci. 2006; 57: 426–445. https://doi.org/10.1111/j.1365-2389.2006.00809.x

16. Dungait, JAJ, Hopkins DW, Gregory AS, Whitmore AP. Soil organic matter turnover is governed by accessibility not recalcitrance. Glob Change Biol. 2012; 18: 1781-1796. https://doi.org/10.1111/j.1365-2486.2012.02665.x

17. Arshad M, Coen G. Characterization of soil quality: physical and chemical criteria. American Journal of Alternative Agriculture. 1992; 7: 25-31. https://doi.org/10.1017/S0889189300004410

18. Tisdall J, Oades J. Organic matter and water-stable aggregates in soils. Eur J Soil Sci. 1982; 33: 141-163. https://doi.org/10.1111/j.1365-2389.1982.tb01755.x

19. Barthès B, Roose E. Aggregate stability as an indicator of soil susceptibility to runoff and erosion; validation at several levels. Catena. 2002; 47: 133–149. https://doi.org/10.1016/S0341-8162(01)00180-1

20. Darboux F, Le Bissonnais Y. Changes in structural stability with soil surface crusting: consequences for erodibility estimation. Eur J Soil Sci. 2007; 58: 1107–1114. https://doi.org/10.1111/j.1365-2389.2007.00906.x

21. Yan, FL, Shi ZH, Li ZX, Cai CF. Estimating interrill soil erosion from aggregate stability of Ultisols in subtropical China. Soil Tillage Res. 2008; 100: 34–41. https://doi.org/10.1016/j.still.2008.04.006

22. Ma Q, Yu WT, Zhao SH, Zhang L. Relationship between water-stable aggregates and nutrients in black soils after reclamation. Pedosphere. 2007; 17: 538–544. https://doi.org/10.1016/S1002-0160(07)60064-2

23. Prager M, Rojas A, Pérez J, Zúñiga O, Gascó J. Actividad y biomasa microbiana como indicadores de materia orgánica en sistemas de cultivo de maracuyá (*Passiflora edulis*) en Toro, Valle del Cauca, Colombia. Acta Agron. 2006; 5: 7-12 https://doi.org/10.15446/acag

24. Di Ciocco CA, Sandler RV, Beatriz-Falco L, Coviella CE. Actividad microbiológica de un suelo sometido a distintos usos y su relación con variables fisicoquímicas. Rev FCA UNCUYO. 2014; 46: 73–85

25. González A, Atencio J, Cubillán K, Almendrales R, Ramírez L, Barrios O. Actividad microbiana en suelos cultivados con plátano (*Musa* AAB subgrupo plátano cv. Hartón) con diferente vigor de plantas. Rev Fac Agron (LUZ). 2014; 31(S1): 526–538.

26. Wanjiru L, Acosta-Martínez V, DeBruyn J, Schaeffer S, Tyler D, Odoi E, et al. Long term tillage, cover crop, and fertilization effects on microbial community structure, activity: implications for soil quality. Soil Biol Biochem. 2015; 89: 24–34. https://doi.org/10.1016/j.soilbio.2015.06.016

27. Chien H, Hou R, Gong Y, Li H, Fan M, Kuzyakov Y. Effects of 11 years of conservation tillage on soil organic matter fractions in wheat monoculture in Loess Plateau of China. Soil Tillage Res. 2009; 106: 85-94. https://doi.org/10.1016/j.still.2009.09.009

28. Whalen JK, Chang C. Macroaggregate characteristics in cultivated soils after 25 annual manure applications. Soil Sci Soc Am J. 2002; 66: 1637–1647. https://doi.org/10.2136/sssaj2002.1637

29. Hernández-Hernández R. Dinámica y manejo de la materia orgánica en suelos de sabanas bien drenadas. Acta Biol Venez. 2010; 28: 69-84

30. Elliott E, Cambardella C. Physical separation of soil organic matter. Agr Ecosyst Environ. 1991; 34: 407-419. https://doi.org/10.1016/0167-8809(91)90124-G

31. McLauchlan KK, Hobbie SE. Comparison of labile soil organic matter fractionation techniques. Soil Sci Soc Am J. 2004; 68: 1616-1625. https://doi.org/10.2136/sssaj2004.1616

32. Wander M. Soil organic matter fractions and their relevance to soil function. In: Magdoff F, Weil R, editors. Soil Organic Matter in Sustainable Agriculture. Boca Raton: CRC Press; 2004. pp. 68-102

33. Belay-Tedla A, Zhou X, Su B, Wan S, Luo Y. Labile, recalcitrant, and microbial carbon and nitrogen pools of a tallgrass prairie soil in the US Great Plains subjected to experimental warming and clipping. Soil Biol Biochem. 2009; 41: 110-116. https://doi.org/10.1016/j.soilbio.2008.10.003

34. Doran JW, Jones AJ, Arshad MA, Gilley JE. Determinants of soil quality and health. In: Lal R, editor. Soil quality and soil Erosion. CRC Press. 1999. pp. 17-36

35. Golchin A, Oades JM, Skjemstad JO, Clarke P. Study of free and occluded particulate organic matter in soils by solid state ^{13}C Cp/MAS NMR spectroscopy and scanning electron microscopy. Aus J Soil Res. 1994; 32: 285–309. https://doi.org/10.1071/SR9940285

36. Sollins P, Swanston C, Kleber M, Filley T, Kramer M, Crow S, et al. Organic C and N stabilization in a forest soil: evidence from sequential density fractionation. Soil Biol Biochem. 2006; 38: 3313-3324. https://doi.org/10.1016/j.soilbio.2006.04.014

37. Puget P, Chenu C, Balesdent JB. Dynamics of soil organic matter associated with particle-size fractions of water-stable aggregates. Eur J Soil Sci. 2000; 51: 595–605. https://doi.org/10.1111/j.1365-2389.2000.00353.x

38. Six J, Elliott ET, Paustian K, Doran JW. Aggregation and soil organic matter accumulation in cultivated and native grassland soils. Soil Sci Soc Am J. 1998; 62: 1367–1377. https://doi.org/10.2136/sssaj1998.03615995006200050032x

39. Christensen BT. Physical fractionation of soil and organic matter in primary particle size and density separates. In: Stewart BA, editor. Advances in Soil Science, vol 20. Springer; 1992. pp. 1-90. https://doi.org/10.1007/978-1-4612-2930-8_1

40. USDA. Keys to Soil Taxonomy. 10th ed. Washington DC: United State Department of Agriculture (USDA) - Natural Resources Conservation Service (NRCS); 2006.

41. Sapucky M. Relaciones entre la productividad del banano (*Musa* AAA) y propiedades físicas y químicas de suelos lacustrinos de la depresión del Lago de Valencia. B.Sc. Thesis, Universidad Central de Venezuela. 2007.

42. Cruz J. Diagnóstico y evaluación del manejo de la fertilización en el cultivo de Banano (*Musa* AAA) en una unidad de producción comercial en el estado Aragua. M.Sc. Thesis, Universidad Central de Venezuela. 2011.

43. Florentino A. Método para evaluar la estabilidad de los agregados de suelo por tamizado en húmedo (Equipo Eijkelkamp) modificado. Laboratorio de Física de suelos. Instituto de Edafología. Facultad de Agronomía. Universidad Central de Venezuela. 2007. 4 p.
44. Meijboom FW, Hassink J, Van Noordwijk M. Density fractionation of soil macroorganic matter using silica suspensions. Soil Biol Biochem. 1995; 8: 1109-1111. https://doi.org/10.1016/0038-0717(95)00028-D
45. Walkley A, Black IA. An examination of Degtjareff method for determining soil organic matter, and a proposed modification of the chromic acid titration method. Soil Sci. 1934; 37: 29-38
46. Heanes DL. Determination of total organic-C in soil by improved chromic acid digestion and spectrophotometric procedure. Commun Soil Sci Plant. 1984; 15: 1191-1213. https://doi.org/10.1080/00103628409367551
47. Bronick CJ, Lal R. Soil structure and management: a review. Geoderma. 2005; 124: 3–22. https://doi.org/10.1016/j.geoderma.2004.03.005
48. Zhang S, Wang R, Yang X, Yun B, Li Q. Soil aggregation and aggregating agents as affected by long term contrasting management of an Anthrosol. Sci Rep-UK. 2016; 6: 39107. https://doi.org/10.1038/srep39107
49. Cabria F, Calandroni M, Monterubbianesi G. Tamaño y estabilidad de agregados y su relación con la conductividad hidráulica saturada en suelos bajo labranza convencional y praderas. Ciencia del Suelo. 2002; 20: 69-79
50. Jastrow J, Miller R. Soil aggregate stabilization and carbon sequestration: feedbacks through organomineral associations. In: Lai R, Kimble J, Follett R, Stewart B, editors. Soil processes and the carbon cycle. CRC Press; 1998. pp. 207-223

51. Zagal E, Rodríguez N, Vidal I, Flores A. La fracción liviana de la materia orgánica de un suelo volcánico bajo distinto manejo agronómico como índice de cambios de la materia orgánica lábil. Agric Tec. 2002; 62: 284-296. http://dx.doi.org/10.4067/S0365-28072002000200011.

52. Caballero R. Fracciones ligera y pesada de la materia macroorganica como fuentes de N y P en suelos de sabanas con diferentes tipos de fertilización. M.Sc. Thesis, Universidad Central de Venezuela. 2011.

53. Barrios E, Buresh RJ, Sprent JI. Organic matter in soil particle size and density fractions from maize and legume cropping systems. Soil Biol Biochem. 1996; 28: 185–193. https://doi.org/10.1016/0038-0717(95)00110-7

54. Christensen BT. Physical fractionation of soil and structural and functional complexity in organic matter turnover. Eur J Soil Sci. 2001; 52: 345–353. https://doi.org/10.1046/j.1365-2389.2001.00417.x

55. Pansu M, Gautheyrou J. Physical fractionation of organic matter. In: Handbook of Soil Analysis: mineralogical, organic and inorganic methods. Pansu M, Gautheyrou J, editors. Springer. 2006. pp. 289-326. https://doi.org/10.1007/978-3-540-31211-6_9

56. Six, J, Paustian K, Elliot ET, Combrink C. Soil structure and organic matter: I. Distribution of aggregate-size classes and aggregate-associated carbon. Soil Sci Soc Am J. 2000; 64: 681-689. https://doi.org/10.2136/sssaj2000.642681x

57. Elliot, ET. Aggregate structure and carbon, nitrogen and phosphorus in native and cultivated soils. Soil Sci Soc Am J. 1986; 50: 627-633. https://doi.org/10.2136/sssaj1986.03615995005000030017x

58. Six J, Merckx R, Kimpe K, Paustian K, Elliot ET. A re-evaluation of the enriched labile soil organic matter fraction. Eur J Soil Sci. 2000; 51:283-293. https://doi.org/10.1046/j.1365-2389.2000.00304.x
59. Galantini J, Suñer L. Las fracciones orgánicas del suelo: análisis en los suelos de la Argentina. AgriScientia. 2008; 25: 41-55. https://doi.org/10.31047/1668.298x.v25.n1.2740
60. Balesdent J, Chenu C, Balabane M. Relationship of soil organic matter dynamics to physical protection and tillage. Soil Tillage Res. 2000; 53: 215-230. https://doi.org/10.1016/S0167-1987(99)00107-5
61. Galantini JA. Contenido y calidad de las fracciones orgánicas del suelo bajo rotaciones con trigo en la región semiárida pampeana. RIA. 2001; 30: 125-146

Fig1

Click here to access/download;Figure;Fig1.tif

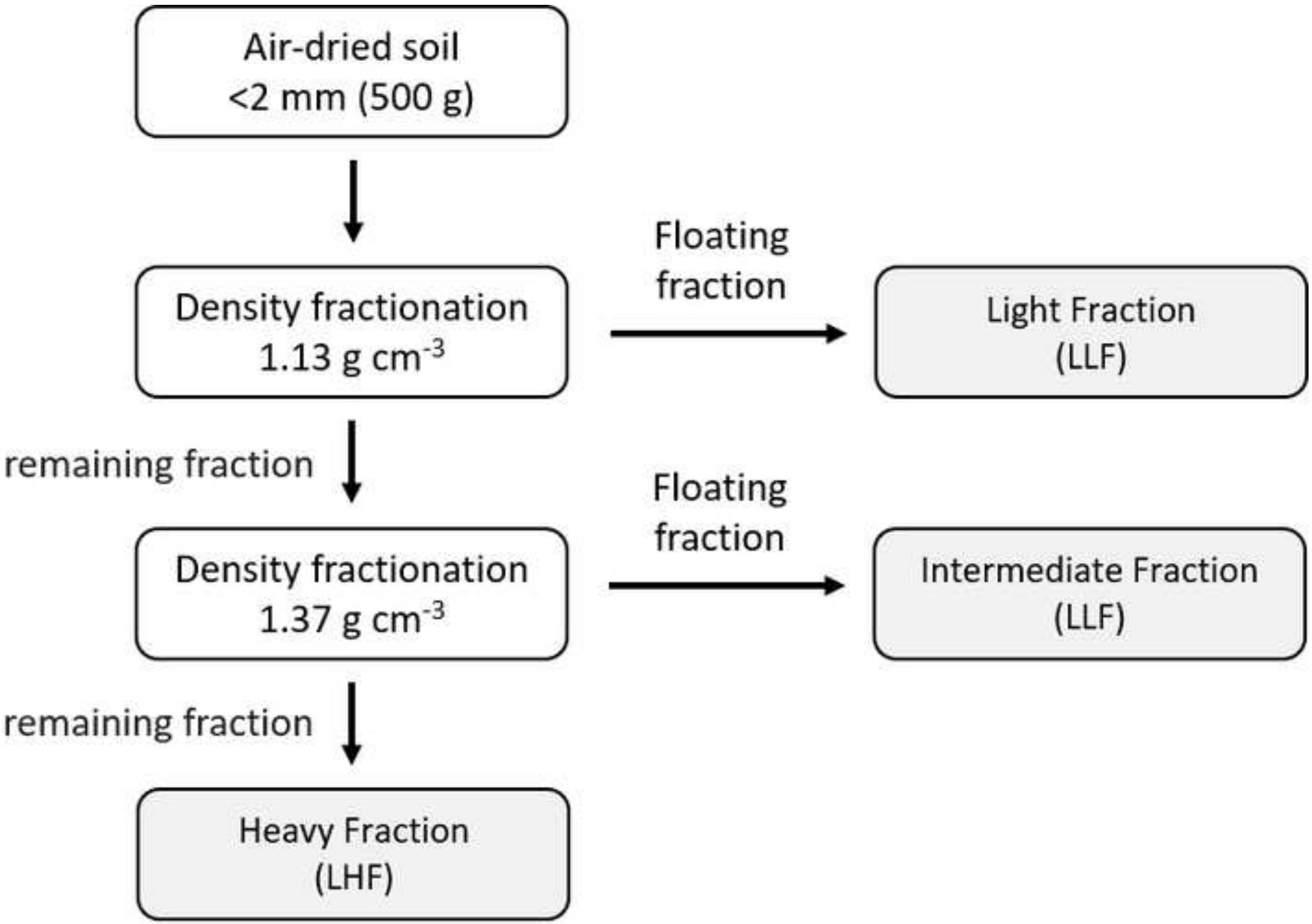

Fig2

Click here to access/download;Figure;Fig2.tif

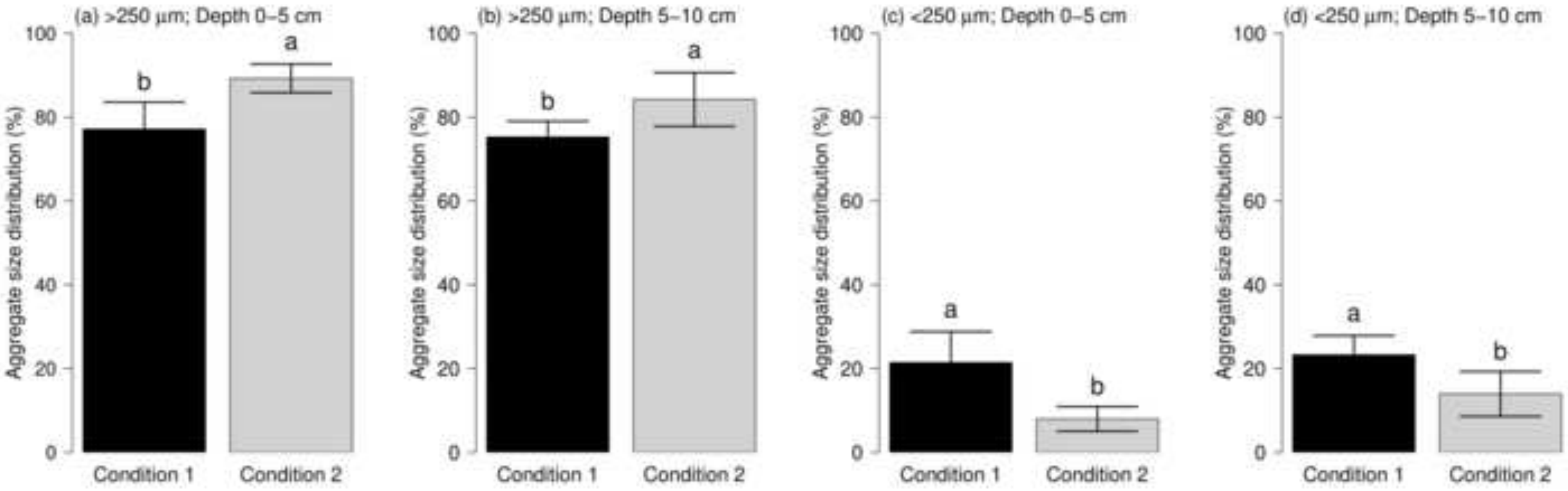

Fig3

Click here to access/download;Figure;Fig3.tif

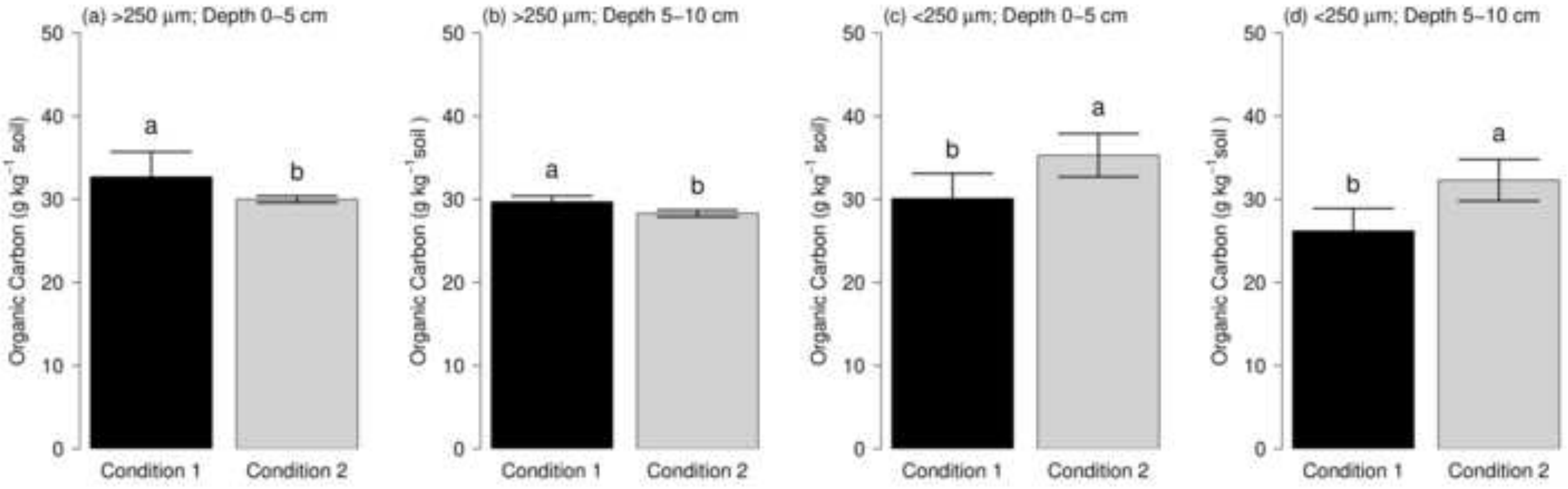

Fig4

Click here to access/download;Figure;Fig4.tif

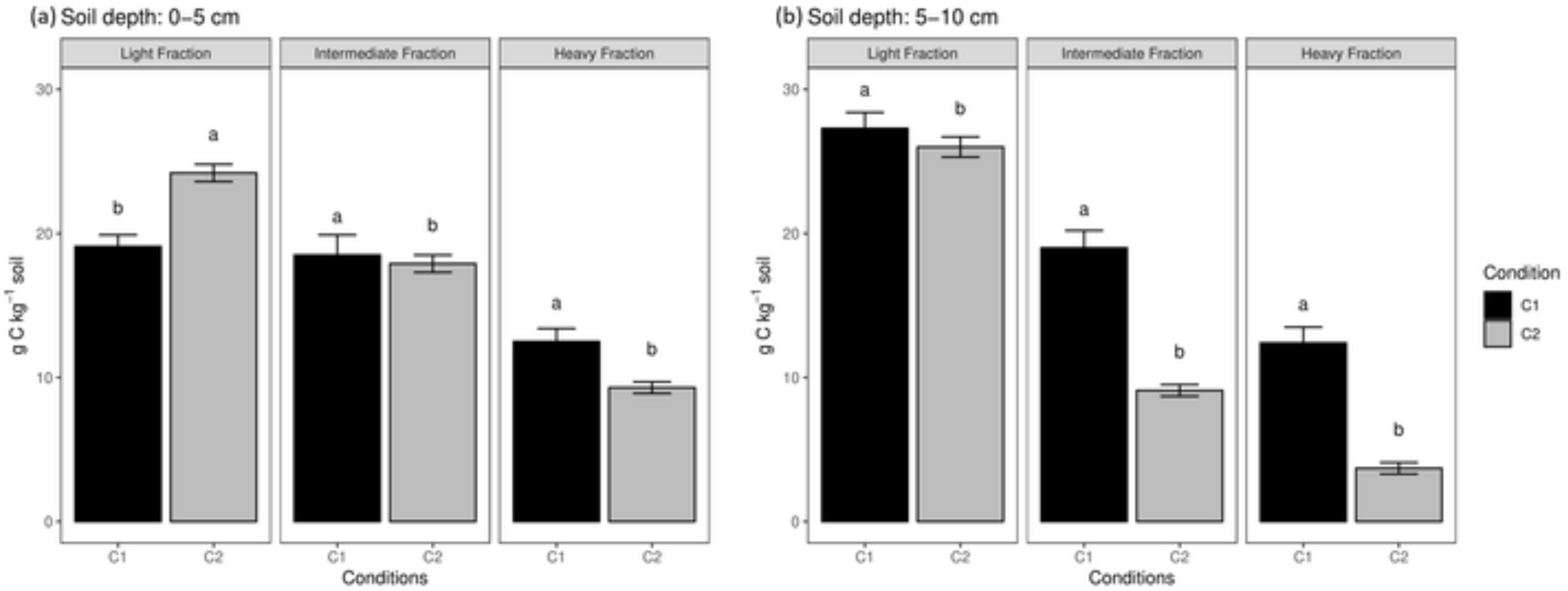

Fig5

Click here to access/download;Figure;Fig5.tiff

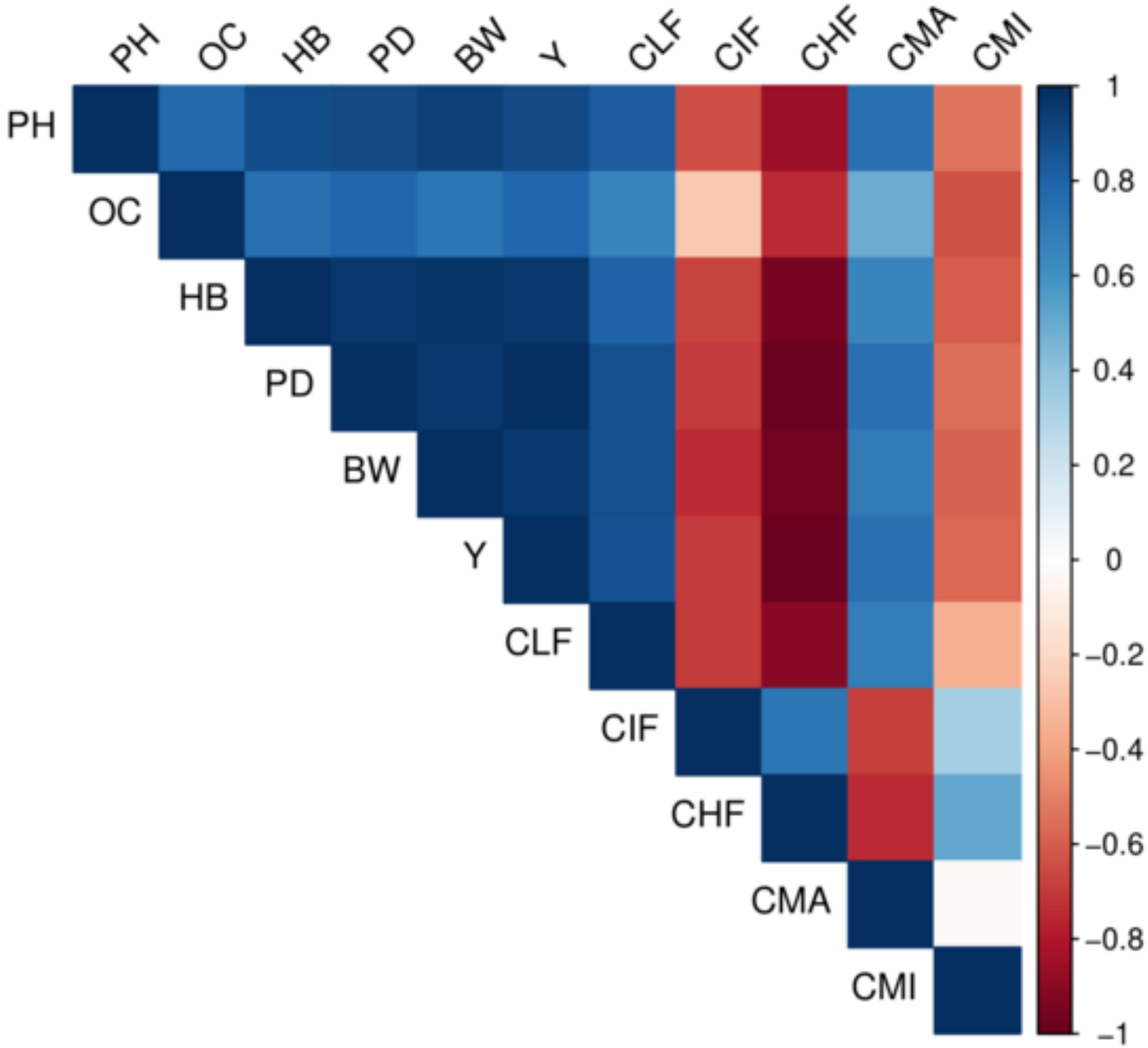

Supplement: Attachment — Submitted filename: PONE-D-20-41023_reviewer.pdf [file pone.0254121.s002.pdf]
