# Supplementary material for: Soil organic carbon, physical fractions of the macro-organic matter, and soil stability relationship in lacustrine soils under banana crop
Source: PLoS One. 2021 Jul 9;16(7):e0254121. doi: 10.1371/journal.pone.0254121 (PMC8270212; doi:10.1371/journal.pone.0254121)
Supplement: Attachment — Submitted filename: Response to Reviewers.pdf [file pone.0254121.s003.pdf]

AGROSAVIA
Corporación colombiana de investigación agropecuaria

Dr. Emily Chenette May 27th, 2021
PLOS ONE Deputy Editor-in-chief
Attention: Dr. Tunira Bhadauria
Academic Editor, PLOS ONE

Dear Editor,

We would like to thank you for the opportunity to revise and resubmit our manuscript PONE-D-20-41023, entitled "*Soil organic carbon, physical fractions of the macro-organic matter, and soil stability relationship in lacustrine soils under banana crop*" for consideration by PLOS ONE journal as original research.

We appreciate the careful review and constructive suggestions. We found the reviewers' comments to be helpful in revising the manuscript and have carefully considered and responded to each suggestion. In most cases, we were successful in incorporating the reviewers' feedback into our revised manuscript. This recent version of the manuscript contains the suggestions of the reviewers and associate editor.

We highlight corresponding changes using Track Charges mode in MS Word. The revision has been developed in consultation with all coauthors, and each author has given approval to the final form of this revision. We have also included a point-by-point response of the major concerns and questions of the reviewers.

On the other hand, we would like to make changes to our financial disclosure, with the following redaction: **"**This work was funded by the Ministry of Science and Technology of Venezuela (www.mincyt.gob.ve), through the Organic Law of Science, Technology, and Innovation (LOCTI, Venezuela) grant 07-01-33-01, project: "Plan de fertilización integral de banano, *Musa* AAA para el aprovechamiento de las fuentes orgánicas en un suelo lacustrino de la depresión del Lago de Valencia". Finca Agropecuaria Punta Larga (www.puntalarga.net) paid for local field workers and reactive chemicals. The funders had no role in study design, data collection and analysis, decision to publish, or preparation of the manuscript.***"***

Thank you for your consideration of this revised manuscript.

Sincerely,

Tatiana M. Rondon S.
Corresponding Author
Research Scientist -AGROSAVIA

Reviewer #1

Thank you for comments. We considered all the specific comments to improve the grammar in the manuscript. Several sentences and words were re-word and highlighted using Track Charges. Figures were included in the recent version of the manuscript.

Reviewer #2

Thank you for comments and suggestions. Introduction was revised and adjusted. In M&M, site description and soil sampling sections were improved to explain the criteria used to separate the banana plots studied. Table 2 was revised and adjusted according to the suggestion.

General Comments

Abstract: revised and adjusted according to the suggestions.
Introduction: Introduction was revised and adjusted, improving the grammar.
Materials and Methods: sentences were revised and modified. Spacing between words were revised and adjusted.
Figures: figures were included in the main text, only for submission. Later, will be delated by the editor or authors.

Comments in the manuscript and responses

N°	Comment	Response
1	Change "biological and chemical" by "biochemical"	Partially accepted. Was included "and their interaction". We think that both processes independently contribute to the stabilization of the soil aggregate.
2	Insert "it"	Done. It was insert in the text.
3	SOM ??	Corrected. The word was changed by "C". the authors refer to the C content.
4	Very old study	-
5	why can't say soil type division as sandy loam and loam,respectively	Accepted. Redaction was improved. This study generated its hypothesis from the results obtained by Sapucky (2007, B.S. Thesis). This author evaluated basic chemical and physical properties, in the same study area, and indicated the relative importance of physical over chemical variables to explain banana productivity; and recommended a detailed SOM and its implication on soil physical properties. At ACORBAT meetings, Rey *et al.* (2006)[a] and Delgado *et al.* (2010)[b] indicated similar results on these soils.

6	Change “starting” by “initial”	Correct. The word was changed.
7	Delete sentence	Accepted. Sentence deleted.
8	Delete sentence	Accepted. Sentence deleted.
9	Change “condition” by “sampling”	Accepted and changed
10	Change by “for each”	Accepted and changed
11	Italic	Done. Corrected in all the text to consistency
12	Insert “method”	Correct. The word was added.
13	should have only two horizontal division .one for 0-5cm and second for 5-10 cm soil. Condition 2 should be written in parentheses.	Accepted. Table was adjusted in the new version of the manuscript.
14	Change by “C content”	Correct. The word was added.
15	Change by “C content”	Correct. The word was added.

[a] Rey JC, Chacín M, Sapuky M, Núñez M, Martínez G, Rodríguez G, Espinoza J, Arturo M, Pocasangre L, Delgado E, Rosales F (2006) Land suitability for banana in Venezuelan soils and its relation to productivity. XVII Internacional Meeting ACORBAT: Banano un negocio sustentable. Joinville. Santa Catarina, Brasil. Nov 15–20.

[b] Delgado EJ, Rey JC, Martínez G, Lobo-Luján D, Sapuky M, Chacin M, Nuñez E, Pocasangre L, Rosales F (2010). Soil properties that determine the vigor of locally produced banana (Musa AAA) in Venezuela. XIX Internacional Meeting ACORBAT Medellín, Colombia, nov 8–12
