# Supplementary material for: Soil organic carbon, physical fractions of the macro-organic matter, and soil stability relationship in lacustrine soils under banana crop
Source: PLoS One. 2021 Jul 9;16(7):e0254121. doi: 10.1371/journal.pone.0254121 (PMC8270212; doi:10.1371/journal.pone.0254121)
Supplement: Attachment — Submitted filename: Cooments.docx [file pone.0254121.s001.docx]

**Comments**

1. **The author conducted a thorough and comprehensive laboratory and field study on** Soil organic carbon, physical fractions of the macro-organic matter, and soil stability relationship in lacustrine soils under banana crop**. The comprehensive data generated from this** experiment will help other scientists working in the similar research area to address the same issue. However, I have identified some moderate level of issues in this manuscript (some editorial and some scientific) which will certainly help to improve the quality of the manuscript. My comments are intended to further improve the manuscript such that it addresses the questions asked properly. I would consider this manuscript for publication if the moderate changes are made and resubmitted. I strongly recommend that author should read the entire manuscript and do not rush on anything before final submission.
2. **Abstract**:

- Please add 1-2 introductory sentences, why this study is important? What is background for this study so that readers can follow it. It is not catchy to start an abstract directly with your methodology approach.
- Please make the concluding remarks more appealing, leave something impactful or some future direction. It is pretty abrupt as conclusion.

3. **Introduction:**

- I think there are several grammatical mistakes in the introduction part, that should be see critically and last paragraph of manuscript should be rewrite.

4. **Materials and methods:**

- Rewrite the sentences in the line 105 to 107 and line 113-116
- Check the spacing between word from line number 120-139

5. **Couldn’t find any figure:** Strongly recommended for the addition of all figures
